# Numerical study of the effect of channel aspect ratio on particle focusing in acoustophoretic devices

**DOI:** 10.1038/s41598-020-76367-w

**Published:** 2020-11-10

**Authors:** L. Spigarelli, N. S. Vasile, C. F. Pirri, G. Canavese

**Affiliations:** 1 Department of Applied Science and Technology, Politecnico di Torino, Corso Duca degli Abruzzi 24, 10129 Turin, Italy; 2 Chilab - Materials and Microsystems Laboratory - DISAT Politecnico di Torino, Via Lungo Piazza d’Armi 6, 10034 Chivasso (Turin), Italy; 3 SynBio Lab, Italian Institute of Technology, Via Livorno 60, 10144 Turin, Italy

**Keywords:** Mathematics and computing, Fluid dynamics, Diagnosis

## Abstract

Acoustophoretic microfluidic devices are promising non-contact and high-throughput tools for particle manipulation. Although the effectiveness of this technique has been widely demonstrated for applications based on micrometer-sized particles, the manipulation and focusing of sub-micrometer ones is challenging due to the presence of acoustic streaming. In this article, our study has the aim to investigate and understand which geometrical parameters could be changed to limit the acoustic streaming effect. We numerically study the well-known rectangular cross section of a microfluidic channel and perform a parametric study of the aspect ratio for several particle sizes. The efficiency of the focusing, is explored for different sized particles in order to identify a trend for which the acoustic streaming does not drastically influence the focusing motion of the particles. The possibility to efficiently separate different solid components in liquid suspensions, i.e. the whole blood, is crucial for all applications that require a purified medium such as plasmapheresis or an increase of the concentration of specific subpopulation as the outcome, such as proteomics, cancer biomarker detections and extracellular vesicles separation.

## Introduction

In the field of focusing and separation of particles, the acoustophoretic approach has gained great interest^1^. This technique is based on the excitation of a standing acoustic wave inside a fluid chamber^2^. If particles are suspended in a medium enclosed in a chamber and an acoustic field is applied, resulting in the formation of a standing acoustic wave, they will experience two effects. The former one is the acoustic radiation force which appears when there is a difference in density and compressibility between the particles and the medium, and the latter is the Stokes drag force, depending on the relative velocity of the particles respect to the acoustic streaming flow^3^. This latter phenomenon represents the main limiting factor of acoustophoretic application for sub-micrometer particles separation^3–5^. Commonly, the acoustic wave is generated by an actuated piezoelectric material which converts electrical voltage to mechanical displacement and strain^6^. Several experimental and theoretical studies are present in literature where a rectangular section with a half-wavelength resonance is considered^3,7–13^. With this assumption, the single node of the standing wave is in the center of the channel, while two anti-nodes are at the sides. This configuration is usually adopted when the aim of the device is to focus particles. When the particle radius is around 1 µm and they present a positive acoustic contrast factor^3^, the acoustic radiation force moves them in the node of the acoustic wave. For particles smaller, with size less than a certain critical radius, the focusing is prevented due to the acoustic streaming^3^. The first theoretical analysis of the boundary-driven acoustic streaming was done by Lord Rayleigh^14^ and then this theory was deepened and investigated by several studies^3,4,8,15–18^. In the recent years, different approaches were numerically explored to overcome the lower size limit^9,19^. In particular, two half-wavelength resonances excited in a channel with a squared section can generate, instead of the common acoustic streaming, a vortex in the center of the section^19^. Despite the disadvantage of obtaining two standing waves instead of one, with this different shape of the vortex, sub-micrometer particles, that feel both the contribution of the acoustic radiation force and the Stokes force, are dragged into a focal point in the center. Another numerical study demonstrates that the simultaneous excitation of two different modes can change the shape and the dimension of the acoustic streaming, effectively decreasing the lower size limit for which the acoustophoretic concentration occurs^9^. An experimental work performed by Hoyos and Castro demonstrates that a pulsed actuation, instead of a steady one, leads to a reduced value of the acoustic streaming, enabling a low, sub-micron particle focusing^20^. A more recent work claimed the possibility to considerably reduce the acoustic streaming by introducing an inhomogeneous fluid in the microfluidic channel^11,12,21^, however this approach leads to a more complex experimental setup. One of the latest studies about the suppression of the acoustic streaming is based on the investigation of shape-optimized section to achieve the lower acoustic streaming influence^22^. Another important result came from a variation of the aspect ratio (AR) of the channel section, as described by Muller et al.^3^. In this work they observed, from numerical results, that by increasing the height of the channel, acoustic streaming does not occur across the entire cross section of the channel. This result could be exploited in devices for applications designed to concentrate the highest number of particles independently of their sizes. Recently, different and pioneering ways to model the acoustophoretic devices were implemented, paving the way to a new 3D approach. Lei et al. applied the limiting velocity finite element method. This strategy allowed them to calculate the driving boundary condition without taking in account of the effects in the boundary layer on a 3D finite fluid volume^23^. A few years later, Bach et al. developed a theoretical description that permitted them to perform numerical simulations of acoustics in a liquid-filled chamber with an arbitrary shape and oscillating elastic solids^24^. In this paper, we chose to extend the model proposed by Muller et al.^3,7^ to numerically analyze different values of AR and its effect on the acoustic streaming over different particles sizes. Since there is a wide number of theoretical and experimental works using the rectangular section investigated by Bruus and coworker, it was adopted as reference system. On the best of our knowledge this is the first systematic study that investigated the effect of different aspect ratios on the focusing performance. Firstly, we changed the height of the channel, keeping constant both the displacement of the actuated walls and the actuating frequency, to obtain a variable acoustic energy density inside the chambers. As second step we investigated the case with acoustic energy density constant, sweeping the displacement of the walls and maintaining the frequency at resonance value of the reference system. This could be interpreted as a voltage sweep in an experimental setup. Then we kept constant the acoustic energy density, fixing the frequency at resonance values and tuning the displacements. Finally, the extension of the section of the channel was set constant for all the aspect ratios and the tests indicated above were repeated. Performing these parametric studies, the principal aim of the work is to improve particle focusing by confining the acoustic streaming far from the focusing region, without adopting an elaborate and complex strategy.

### Governing equations

In this section, we present the governing equations used in the model. As described firstly by Muller et al.^3,25^, the perturbation theory is used to derive first order and second-order acoustic fields in a compressible Newtonian liquid. Similarly, we considered only the fluidic domain, thus the high acoustic impedance solid material, typically glass-silicon, is considered as the ideal boundary for hard rigid walls. For estimating the quantitative difference in assuming an adiabatic system instead of considering all the thermoviscous effect, we performed tests with the model proposed more recently by Muller et al.^26^—the results are shown in Table 1. The resulting fields do not present remarkable differences. Since the differences were low and to reduce the computation time, we chose to proceed the study by considering a rectangular cross section 380 µm wide (W) and 160 µm high (H) with the adiabatic assumption. We considered a homogeneous, isotropic fluid with unperturbed thermodynamic equilibrium state denoted as zeroth order which corresponds to a temperature *T*_*0*_, density *ρ*_*0*_, pressure *p*_*0*_ and quiescence, ***v***_***0***_ = 0. If the entropy is conserved due to the adiabatic assumption, the thermodynamics of the system could be described by only the pressure *p.* The changes from the equilibrium in density *dρ* could be described as related to the change in pressure *dp* through the isentropic compressibility *k*_*s*_, as follows^3^:

1 $$d\rho = \rho k_{s} dp$$

**Table 1:** Estimation of the error done considering adiabatic assumption. Results obtained considering thermoviscous effects are set as reference values.

Variable	Error
vx1	0.2494 [m s^−1^]
vy1	1.2560 × 10^–4^ [m s^−1^]
p1	0.3751 [MPa]
p2	81.5837 [Pa]
vx2	4.0135 × 10^–4^ [m s^−1^]
vy2	1.7834 × 10^–4^ [m s^−1^]

Using the standard perturbation theory all the fields could be write as series of orders (zeroth, first and second order respectively indexed with *0*, *1* and *2*):

2.1 $${\varvec{v}}\boldsymbol{ }=\boldsymbol{ }{{\varvec{v}}}_{1}+\boldsymbol{ }{{\varvec{v}}}_{2},$$

2.2 $${\varvec{p}}\boldsymbol{ }={p}_{0}\boldsymbol{ }+\boldsymbol{ }{p}_{1}+\boldsymbol{ }{p}_{2}$$

All first-order fields are assumed as time dependent with harmonic behavior:

3 $${g}_{1}({\varvec{r}},t) = Re\{ {g}_{1}({\varvec{r}})\boldsymbol{ }{e}^{-i\omega t}\},$$

where ${g}_{1}$ is used to indicate a generic first-order field, ω = 2π*f* = *c*_*0*_*π/W *is the angular frequency, with the frequency denoted by *f* and c_0_ is the speed of sound in the fluid defined as *c*_*0*_ = *1/(k*_*s*_* ρ*_*0*_*)*^*0.5*^*.* From Eq. (3), it is simple to define that the time derivative of a first-order field becomes *∂*_*t*_${g}_{1} = -{i\omega g}_{1}$. First-order equations are obtained neglecting all the other terms, thus the continuity equation and the momentum equation become

4.1 $${k}_{s}{\partial }_{t}{p}_{1} = -\nabla \cdot {{\varvec{v}}}_{1}$$

4.2 $${\rho }_{0}{\partial }_{t}{{\varvec{v}}}_{1} = \nabla \cdot{({\varvec{\tau}}}_{1}- {p}_{1}{\varvec{I}})$$

where **τ**_1_ is the first-order shear stress tensor and is defined as follows:

5 $${{\varvec{\tau}}}_{1}={\mu }_{0}\left[\nabla {{\varvec{v}}}_{1}+\nabla {{\varvec{v}}}_{1}^{T}\right]+ ({\mu }_{0}^{\mathrm{b}} - \frac{2}{3} {\upmu }_{0})\nabla \cdot {{\varvec{v}}}_{1}{\varvec{I}}$$

with µ_0_ the dynamic viscosity, µ_0_^b^ is the bulk viscosity, ***I*** is the identity matrix and T is referred to the transpose. Considering the frequency domain, the first-order equations can be simplified by changing the time derivative. Thus, Eqs. (4.1) and (4.2) becomes:

6.1 $$-i\omega {k}_{s}{p}_{1} +\nabla \cdot
{{\varvec{v}}}_{1} = 0,$$

6.2 $${i\omega \rho }_{0}{{\varvec{v}}}_{1} + \nabla \cdot {({\varvec{\tau}}}_{1}- {p}_{1}{\varvec{I}}) = 0$$

The time averaged second-order equations take the following form, where squared brackets denote the time average over one full oscillation period.

7.1 $$\nabla \cdot [\langle {{\varvec{v}}}_{2}\rangle {\rho }_{0} + \langle {\rho }_{1}{{\varvec{v}}}_{1}\rangle ]= 0$$

7.2 $$\nabla \cdot [\langle {{\varvec{\tau}}}_{2}\rangle - \langle {p}_{2}\rangle I - {\rho }_{0} \langle {{\varvec{v}}}_{1}{{\varvec{v}}}_{1}\rangle ]= 0$$

where $\langle {{\varvec{\uptau}}}_{2}\rangle$ is given by:

7.3 $$\langle {{\varvec{\tau}}}_{2}\rangle ={\upmu }_{0}[\nabla \langle {{\varvec{v}}}_{2}\rangle +{\nabla \langle {{\varvec{v}}}_{2}\rangle }^{T}]+ ({\mu }_{0}^{b} - \frac{2}{3} {\upmu }_{0})\nabla \cdot \langle {{\varvec{v}}}_{2}\rangle {\varvec{I}}$$

It is important to notice that the time average of the product of two harmonic first-order fields is calculated as follows, where the * is the complex-conjugated:

8 $$\langle {g}_{1}{g}_{1}\rangle = 0.5Re[{({g}_{1})}^{*}{g}_{1}]$$

The time averaged acoustic energy density is expressed as^16^:

9 $$\langle {E}_{ac}\rangle ={\int }_{V}0.5{k}_{s}\langle {{\varvec{p}}}_{1}{{\varvec{p}}}_{1}\rangle + 0.5{\rho }_{0}\langle {{\varvec{v}}}_{1}\cdot {{\varvec{v}}}_{1}\rangle dV$$

### Numerical model and boundary conditions

As mentioned above, we define only the fluid inside the channel as the control volume. For less computing demand, a 2D rectangular cross section was considered. The dimensions of the initial section of the channel were taken from Muller et al.^3^, thus the width is 380 µm and the height is 160 µm. This corresponds to an AR, calculated as the ratio between the height and width of the channel, of 0.42. For exciting the first mode, we set the frequency equal to the channel resonance frequency of 1.9669 MHz. The simulations are implemented in COMSOL Multiphysics (version 5.4, COMSOL Inc.), the equations are implemented as PDE weak form, and the particle motion is computed using the built-in module of Particle Tracing for Fluid Flow. Since the right propagation of the bulk acoustic waves inside the fluid requires a device with high acoustic impedance material (i.e. silicon/glass device), it is reasonable to assume the top and the bottom walls as rigid, without any displacement. The actuation on the right and left walls is modeled adding a harmonically oscillating boundary condition of the first order velocity, while for the second order velocity field the zero-mass flux through these walls is set as boundary condition^26^, as expressed in Eqs. (10.2), (10.3), and (10.4).

10.1 $$T = { }T_{0} = 25{ }^\circ C\;\;\;\;{\text{on}}\;\;{\text{all}}\;\;{\text{ walls}},$$

10.2 $${\mathbf{v}} = { }0\;\;\;\;on\;\;all\;\;walls,$$

10.3 $${\mathbf{n}} \cdot {\text{v}}_{1} = {\text{ v}}_{{{\text{bc}}}} \left( {{\text{x}},{\text{y}}} \right){\text{e}}^{{ - {\text{i}}\omega {\text{t}}}} \;{\text{on}}\;\;{\text{ the }}\;{\text{right}}/{\text{left }}\;{\text{walls}},$$

10.4 $${\mathbf{n}} \cdot {\text{v}}_{2} = - \frac{{{\uprho }_{1} \left( {{\mathbf{n}} \cdot {\text{v}}_{1} } \right)}}{{{\uprho }_{0} }}{\text{ on}}\;{\text{ the}}\;{\text{ right}}/{\text{left}}\;{\text{ walls}},$$

where *n* is outward normal vector to the surface, ${\mathrm{v}}_{\mathrm{bc}}= \omega {l}_{0}$, with ${l}_{0}$ = 0.1 nm which is a representative displacement^3,27^ consistent with other numerical works^3,26^ and with experiments^8^. For the pressure, a standard null flux boundary condition is maintained at the walls. However, it is necessary to use the Lagrange multiplier which forces the second-order pressure average to zero and achieves convergence. All the parameters for water and for polystyrene particles are listed in Table 2 and they are taken from van’t Oever et al.^9^. These parameters are fundamental for the right computation of the acoustic field and forces acting on the particles in the particle tracing.

**Table 2:** Parameters used for pure water and polystyrene particles at T = 25 °C.

Parameter	Symbol	Value
**Water**		
Density^1^	ρ_0_	998 [kg m^−3^]
Speed of sound^1^	c_0_	1497 [m s^−1^]
Shear viscosity^2^	µ_0_	0.890 [mPa s]
Bulk viscosity^3^	${\mu }_{0}^{b}$	2.485 [mPa s]
Specific heat capacity^1^	C_p_	4181 [J(kg K)^−1^]
Heat capacity ratio^1^	Γ	1.011
Thermal conductivity^4^	k_th_	0.6065 [W(m K)^−1^]
Isentropic compressibility^5^	k_s_	448 [TPa^−1^]
Thermal expansion coeff.^2^	α_p_	2.573 $\times$ 10^–4^ [K^−1^]
**Polystyrene**		
Density^8^	ρ_ps_	1050 [kg m^−3^]
Speed of sound	c_ps_	2350 [m s^−1^]
Compressibility	k_ps_	249 [TPa^−1^]
Poisson’s ratio^9^	σ_ps_	0.35
Heat capacity^10^	C_p,ps_	1220 [J(kg K)^−1^]
Heat capacity ratio^11^	γ_ps_	1.04
Thermal expansion coeff.^11^	α_p,ps_	2.09 $\times$ 10^–4^ [K^−1^]
Thermal conductivity^12^	k_th,ps_	0.140 [ W(m K)^−1^]
Isentropic compressibility^6^	k_s,ps_	238 [TPa^−1^]
Speed of sound^6^	c_s,ps_	2350 [m s^−1^]
Transverse speed of sound^7^	c_t,ps_	1068 [m s^−1^]

The list below the table reports the references from which some parameters are taken.

^1^From polynomial fit from Ref.^26^, based on data from Ref.^28^.

^2^From polynomial fit from Ref.^26^, based on data from Ref.^29^.

^3^From polynomial fit from Ref.^26^, based on data from Ref.^30^.

^4^From polynomial fit from Ref.^26^, based on data from Ref.^31^.

^5^From Ref.^16^.

^6^From Ref.^9^.

^7^From Ref.^9^, taken from^32^.

^8^From Ref.^3^.

^9^From Ref.^9^, taken from^33^.

^10^From Ref.^9^, taken from^34^.

^11^From Ref.^32^.

^12^From Ref.^9^, taken from^35^.

### Mesh convergence analysis

**Figure 1:**
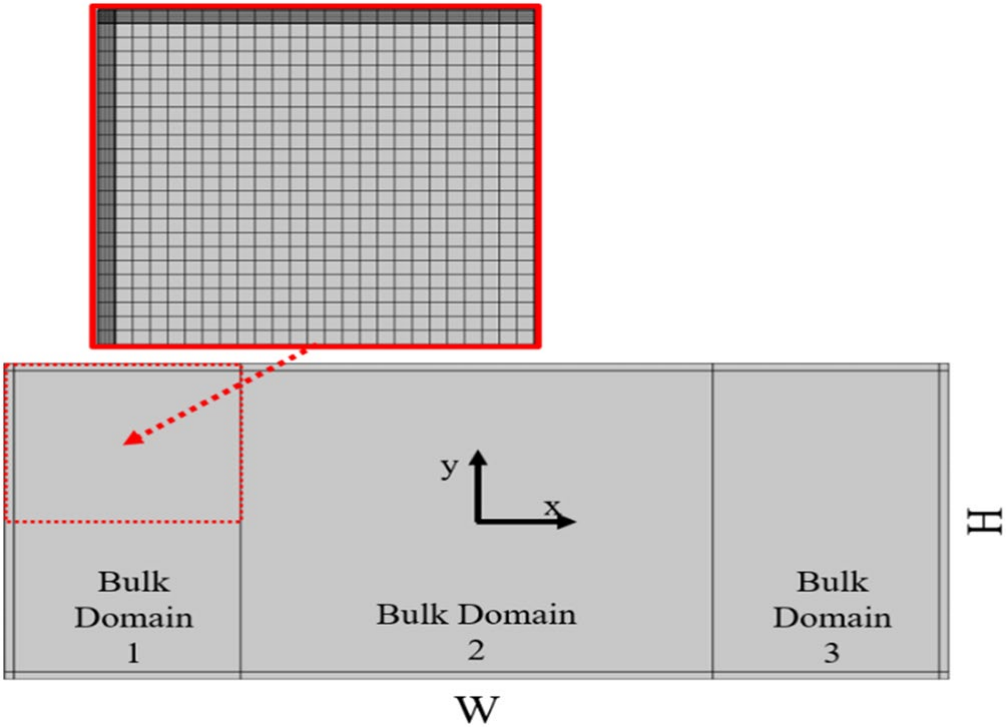
Schematic image of the rectangular cross section (W × H) of the channel chose as reference system. The control volume is divided in three bulk domains and one boundary domain with height w_bk_. A zoom of the red squared zone is reported to show the mesh elements.

The whole rectangular section of the channel was divided in four subdomains as shown in Fig. 1, three considered as bulk fluid and one as boundary fluid domain with height w_bd_, set equal to $10{\delta }_{s}$, where ${\delta }_{s}$ is the viscous boundary layer defines as:

11 $${\delta }_{s}=\sqrt{\frac{{2\mu }_{0}}{{\rho }_{0}\omega }}$$

**Figure 2:**
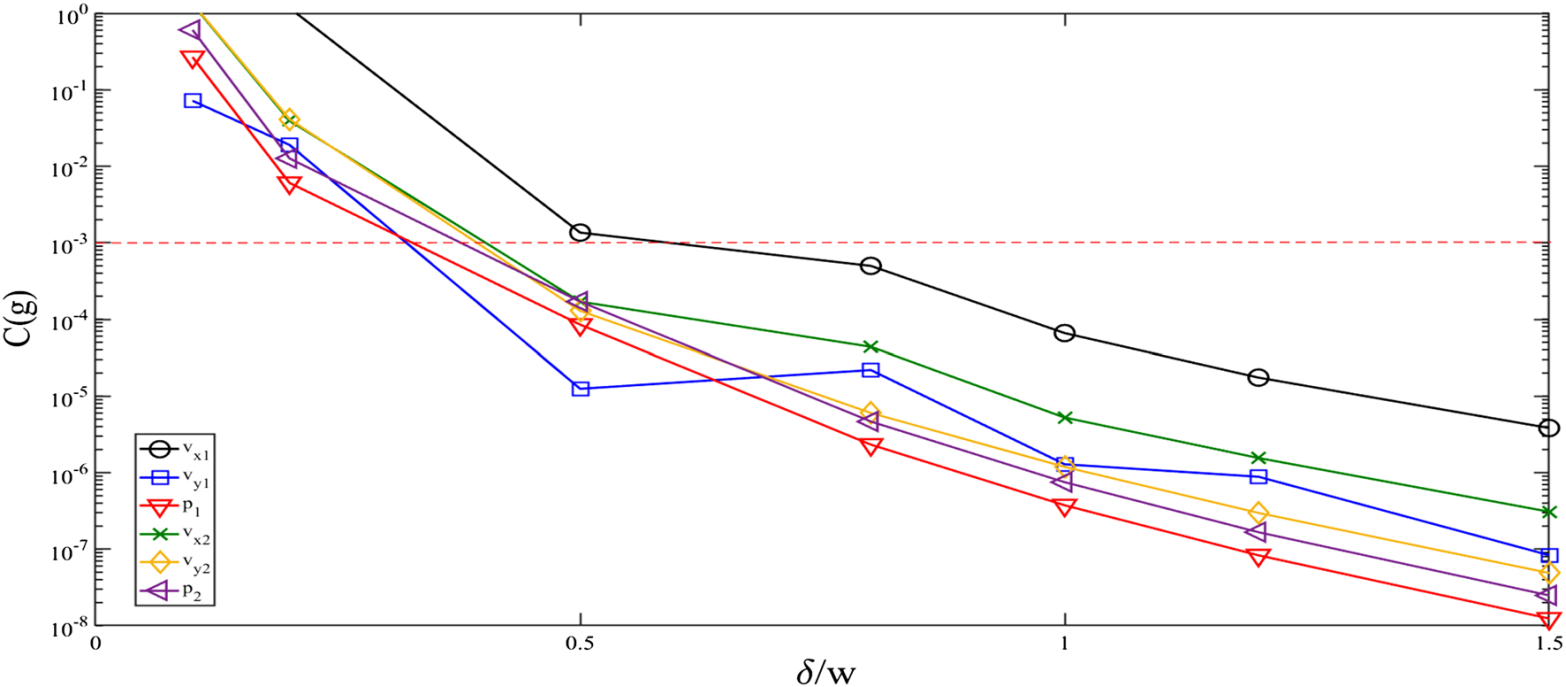
Mesh convergence analysis for different element sizes in the boundary domain.

The viscous boundary layer for a chamber filled with water is typical about 0.4 µm for a frequency of 2 MHz. The bulk domain, that goes from − W/2 + w_bd_ to W/2 − w_bd_, is discretized using a Free Quad mesh with a maximum element size w equal to w_bd_, while the boundary domain is divided, using a mapped mesh, in N elements. As shown in Fig. 2, we performed a mesh convergence analysis with varying N so that the relative convergence parameter measured $\mathrm{C}(\mathrm{g})$ is lower than 1 × 10^–3^. It is estimated by considering a general solution for g and the reference solution g_ref_, calculated using the finest mesh, as follows:

12 $$C\left(g\right)= \frac{\sqrt{\int {(g-{g}_{ref})}^{2}dxdy}}{\sqrt{\int {g}_{ref}^{2}dxdy}}.$$

The reference mesh is chosen to maintain an element size in the bulk that is equal to w and with N = 20, which corresponds with an element size inside the boundary bulk that is equal to 0.5 ${\delta }_{s}$ (or ${\delta }_{s}$/w = 2) . In Fig. 2, the results from the convergence analysis are shown and we chose to use N = 8 for all the further tests, which correspond to a value of ${\delta }_{s}/\mathrm{w}$ = 0,8. With these settings, the total number of elements is approximately 6 × 10^3^. We focused our attention to the domain that is defined between -W/4 and W/4, which hereinafter we refer to as “central domain”.

### Particle tracing

After the computation of the entire acoustic field, we can use the results to calculate all the forces experienced by the particles. In a finite domain, the streaming can be computed and the total force experienced by a particle suspended in a medium is the sum of the acoustic radiation force and the streaming-induced Stokes drag force^32^. The balance between acoustic radiation force and drag force is strictly dependent on the particle size. In fact, the acoustic radiation force is function of the cubic of the radius (Eq. 13) and the drag force is linearly dependent on the radius (Eq. 15). This means that for larger sized particles the acoustic radiation force will be dominant, and they will be focused at the node/antinode as a function of their acoustic contrast factor. Meanwhile, if submicrometric particles are considered, the drag force has a higher magnitude compared to acoustic radiation force, thus these are forced to follow the well-known four-vortex pattern^3,12,24^ and they cannot be focused. In this work, we modelled the acoustic radiation force using the formula obtained by Karlsen et al.^9,32^, which has the form expressed in Eq. (13), where *a* is the particle radius and C_M_ and C_D_ are used for the monopole and dipole scattering terms^9,32^ (for a complete explanation of the terms refer to^9^).

13 $${F}_{rad}=-\pi {a}^{3}\left[\frac{2}{3}{k}_{s}\langle {C}_{M}{p}_{1}\nabla {p}_{1}\rangle -{\rho }_{0}\langle {C}_{D}{{\varvec{v}}}_{1}\cdot {{\varvec{v}}}_{1}\rangle \right],$$

14.1 $${C}_{M}= \frac{{C}_{M1}+{C}_{M2}H}{1 + {C}_{M3}H}$$

14.2 $${C}_{D}= \frac{{C}_{D1}\left(1-G\right)}{{C}_{D1} +3\left(1-G\right)}$$

The time-averaged Stokes drag force is defined through the difference between the time averaged acoustic streaming ${{\varvec{v}}}_{2}$ and the particle velocity ${{\varvec{v}}}_{p}$, as follows:

15 $${F}_{drag}=6\pi {\mu }_{0}a\left(\langle {{\varvec{v}}}_{2}\rangle -{{\varvec{v}}}_{p}\right).$$

**Figure 3:**
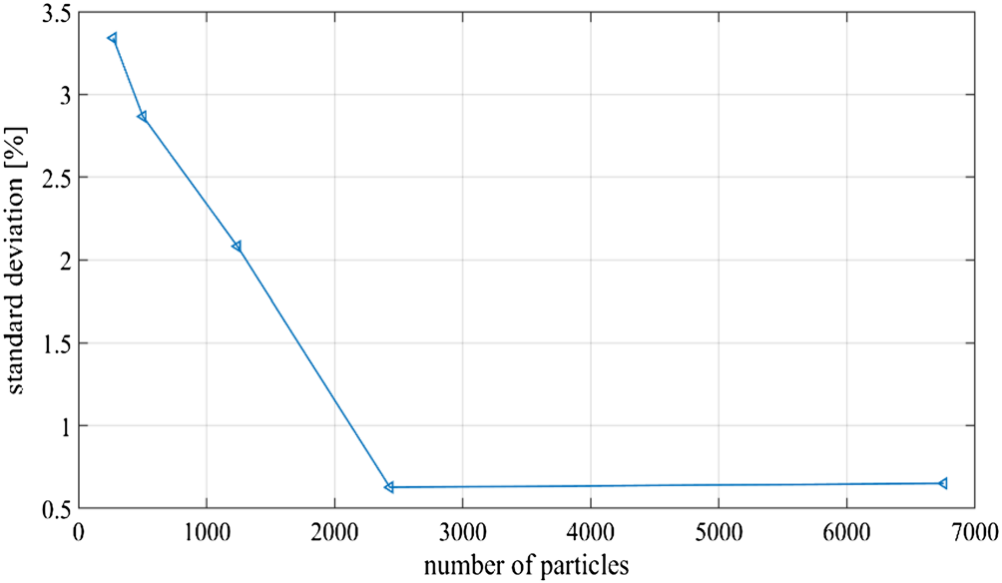
Standard deviation of the fraction of particles collected in subdomain 2.

For the particle tracing, the built-in COMSOL interface Particle Tracing for Fluid Flow is used. With a Newtonian formulation and assuming a time dependent solver, the equation of the balance of the forces is:

16 $$\frac{d(m{{\varvec{v}}}_{p})}{dt}= {F}_{T}$$

where m is the mass of the particle, ${{\varvec{v}}}_{p}$ is the particle velocity and ${F}_{T}$ is the sum of the forces acting on the particles. The left side term expresses the acceleration of the particles which has a faster time scale respect to the terminal velocity of the particles. In fact, considering a micrometer-sized particle accelerated by the drag force ${F}_{drag}$, the characteristic unsteady time can be approximated as ${\tau }_{unsteady}\approx (2/9){a}^{2}{\rho }_{p}/{\mu }_{0} \approx 1 \mathrm{\mu s}$. On the other hand the characteristic steady time, with a terminal velocity ${{\varvec{v}}}_{p}$ is ${\tau }_{steady} \approx 1 ms$ , obtained from ${{\varvec{v}}}_{p} = {F}_{rad}/(6\pi {\mu }_{0}a)$. So, it is possible to consider just the balance between acoustic radiation force and the Stokes drag force acting on a particle when computing the terminal velocity and the corresponding position evolution over time. We considered particles, released from a regular grid in the whole domain, with an initial velocity equal to 0. The interactions between particles are not considered. Particles with radius *a* equal to 250 nm, 500 nm, 750 nm, 1 µm and 2 µm respectively, were studied. We observed that the release grid spacing, thus the concentration of particles, influenced the results, especially for smaller particles. Therefore, we performed some tests for the most critical situation, i.e. AR of 0.42 and particles radius of 250 nm (for further information see Supplementary Information, Fig. S1). As expected, the fraction of particles collected in the bulk subdomain 2 presented greater oscillations over time with decreasing particle number. Analyzing the standard deviation of the fraction of particles focused for a decreasing grid space, we found a limit number of particles for which the results did not change. Accordingly, we chose to keep constant the spacing of the grid at 5 µm, which corresponds to a standard deviation under 1%, as shown in Fig. 3. This concentration of particles is compatible with the assumption of neglecting the hydrodynamic interaction between micron sized particles^19^.

## Results

We investigated different parameters in order to cover several experimental conditions commonly experienced within an acoustophoretic device (i.e. sweeping frequency or voltage for achieve the required acoustic energy density inside the channel). A schematic summary of how the study was structured is reported in Table 3.

**Table 3:** Summary of the structure of the present study, where W is the width of the channel and S is the cross-sectional area of the channel defined as W × H.

E_ac_	f	l_0_
**W constant**		
Variable	Constant at 1.9669 MHz	Constant at 0.1 nm
Constant at 106 Pa	Constant at 1.9669 MHz	Variable
Constant at 106 Pa	Constant at resonance frequency	Variable
**S constant**		
Constant at 106 Pa	Variable	Constant at 0.1 nm
Constant at 106 Pa	Constant at resonance frequency	Variable

### Varying the AR by maintaining the channel width fixed

#### Variable acoustic energy density and constant actuation parameters

**Figure 4:**
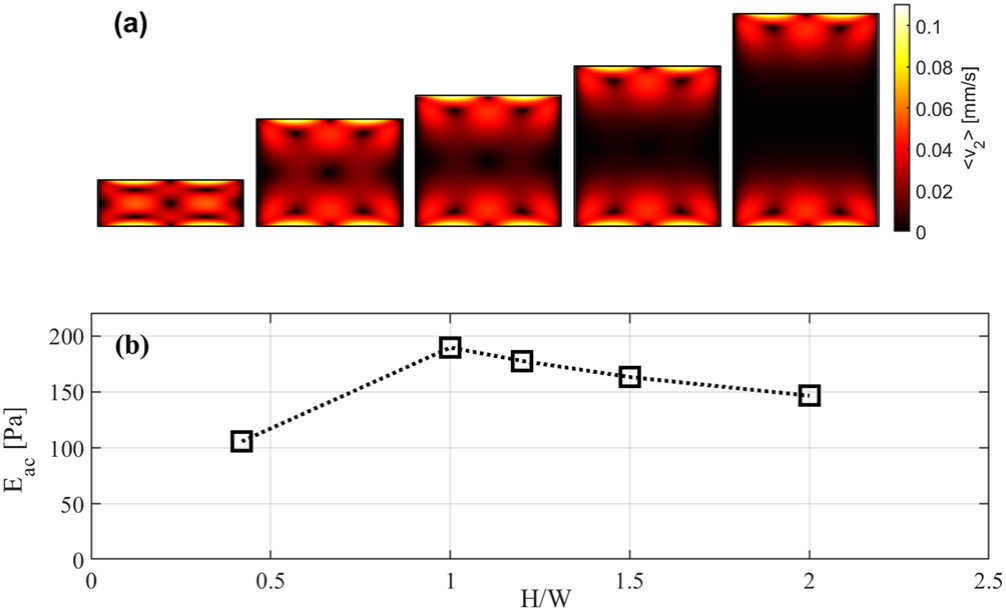
(**a**) Acoustic streaming contour plots for the 5 used aspect ratios (from left to right 0.42, 1, 1.2, 1.5, 2 respectively). (**b**) Acoustic energy density as function of the aspect ratio.

**Figure 5:**
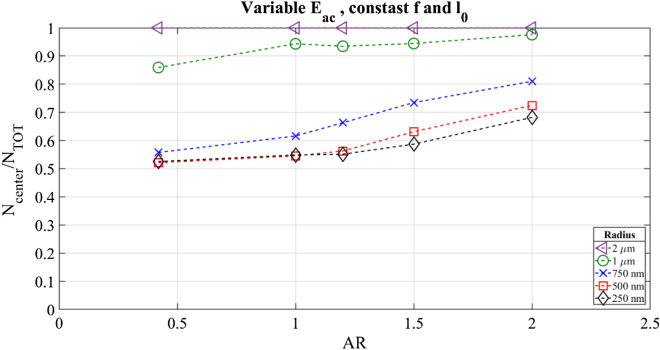
Fraction of particles in the central bulk subdomain, compared to the total number released, as function of their radius. These results were recorded at t = 16 s after particles release. In this case, the wall displacement was kept constant at 0.1 nm.

We started by analyzing a parametric sweep in height, maintaining constant the displacement of the side walls l_0_ to 0.1 nm and the width W of the channel at 380 µm. The adopted aspect ratios were 0.42, 1, 1.2, 1.5 and 2, respectively. As a first step, the frequency was kept constant at 1.9669 MHz, which corresponds to the resonance frequency of the reference system, and the corresponding acoustic energy densities, calculated with Eq. (9), are reported in Fig. 4b. The acoustic streaming goes to 0 in the central part of the section as the aspect ratio value increases, as shown with the contour plots reported in Fig. 4a. The reason for this behavior was already stated by Muller et al.^3^. The zone affected by acoustic vortex decreases in size when the top and bottom walls are moved away from each other. The influence of boundary walls is localized and leads to a “free zone” in the middle of the channel section where the acoustic radiation force becomes dominant for extended time. The acoustic energy density presents a maximum for a squared section and it decreases when the aspect ratio becomes greater, with a minimum obtained with AR equal to 0.42. This contrasting behavior could be explained analyzing Eq. (9). Since the acoustic energy density is calculated over the volume, a higher cross section leads to reduced energy density. On the other hand, by increasing the height of the oscillating walls, a greater mechanical energy enters the system. Considering the number of particles collected in the center for all the aspect ratios, we found that this value started to oscillate after few seconds for lower AR. Therefore, the results for the first 16 s were analyzed, where the higher performance condition of focusing was found (see Supplementary Information, Fig. S2). Figure 5 shows an increasing of the efficiency of particle focusing at the center of the channel for higher aspect ratios. Great differences appear for particles with radius under a micron, for which the focusing fractions change by 15–20%. The purple line represents the percentage of particles with 4 µm diameter. In this condition, the acoustic radiation force is totally dominant with respect to the drag force which maintains all the particles within the center of the channel. At first sight, higher aspect ratios permit to overcome the size limit for which bulk acoustic wave devices can focus particles.

#### Fixed acoustic energy density varying the walls displacement

**Figure 6:**
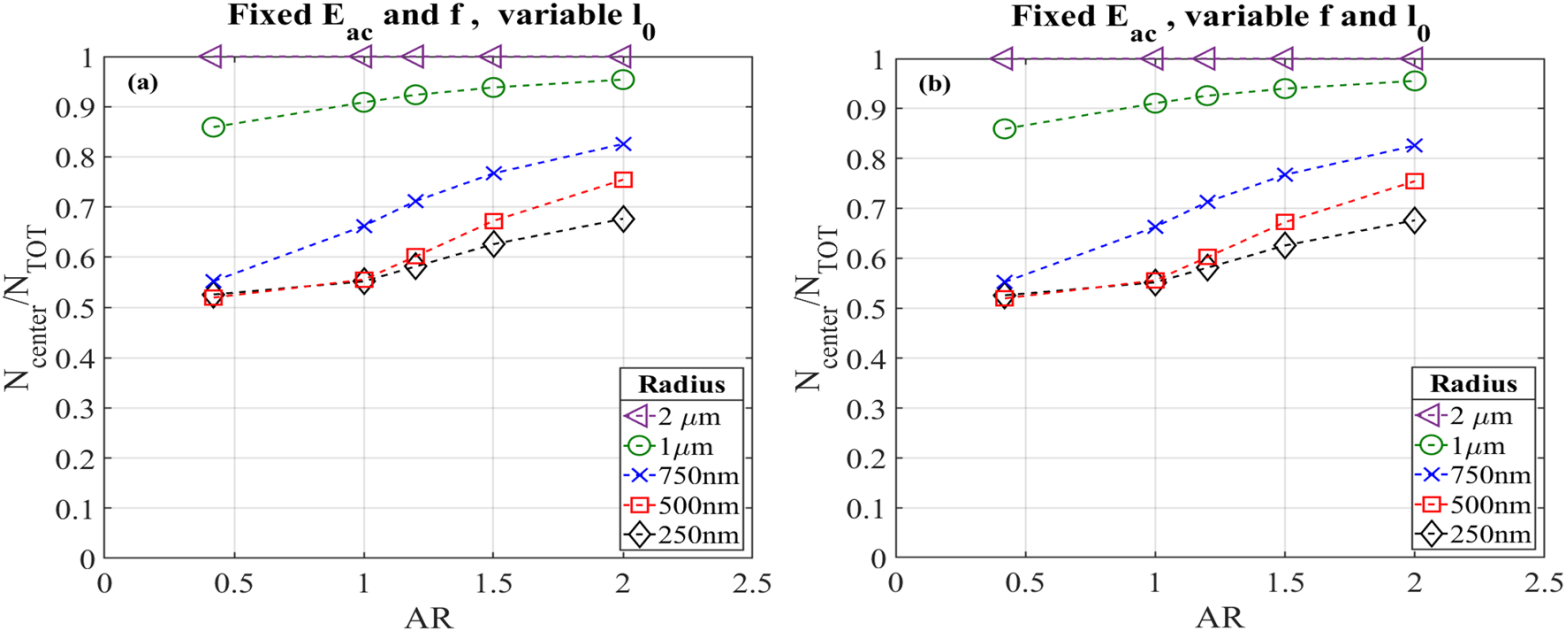
Fractions of particles in the central subdomain, compared to the total number released, as function of their radius. These results are recorded at t = 16 s after particles release. In this case the acoustic energy density is kept constant around 106 Pa varying the displacement l_0_. (**a**) The frequency was kept constant at 1.9669 MHz, the resonance frequency of the reference system. (**b**) The frequency was changed for each AR to the resonance value and the displacement was varied obtaining an acoustic energy density constant.

After obtaining the results and keeping constant all the actuation parameters, we varied the displacement of the walls to maintain constant the acoustic energy density inside the chamber at the same frequency of 1.9669 MHz. A parametric sweep on the displacement l_0_ for all the aspect ratios was performed to achieve an E_ac_ around 106 Pa, as calculated for the reference system (AR equal to 0.42). Results obtained from the particle tracing are reported in Fig. 6a. As performed above, fractions of particles collected in the center of the channel were calculated at 16 s after the release. In contrast to previous results, the increase of percentages of focused particles is less marked. However, a trend in the fraction of particles concentrated as a function of the aspect ratio is appreciable. In this case, the percentage of particles with radius of 500 nm exhibits an increased focusing with high AR. Furthermore, particles with 750 nm of radius present a greater focusing also for low aspect ratios.

#### Fixed acoustic energy density at resonance frequency varying the wall displacement

The combination of resonance frequency and the voltage is important to ensure the optimal transmission of mechanical energy into the device. In order to understand if the condition of resonance could influence the results, we performed the same study as in the section above but set the frequency at resonance values optimized for each aspect ratios (see Fig. 6b). There is not an evident difference between this approach and the one proposed in the previous sub-section with variable wall displacement and the frequency set as constant. Therefore, it is reasonable to assume that the condition of resonance is not essential (even if it is experimentally convenient exploit this favorable energy condition) when keeping constant the acoustic energy density, since the required mechanical energy could be provided to the system through greater/lower voltage (in the experimental limit).

### Varying the AR with constant cross-sectional area

If the height of the channel was varied for each aspect ratio, while the width was maintained constant, the area of the channel cross section changed. Obviously, this changes the resonance and the energy density harvested and transformed by the system. We analyzed the condition of constant area (by keeping fixed the cross section at the value of 60,800 µm^2^ corresponding to the reference channel 380 µm width and a 160 µm height), investigating the same values of AR. The width and height used for the following sections are reported in Tables 4 and 5, respectively.

**Table 4:** Summary of height, width and the corresponding frequencies used for the study for fixed acoustic energy density, wall displacement and cross-sectional area.

H [µm]	W [µm]	f [MHz]	AR
247	247	3.0226	1
270	225	3.3177	1.2
302	201	3.7132	1.5
349	174	4.2881	2

**Table 5:** Summary of height, width and the corresponding resonance frequency and walls displacement used for fixed acoustic energy density and cross-sectional area.

H [µm]	W [µm]	f_res_ [MHz]	AR	l_0_ [m]
247	247	3.028	1	3.460e^−11^
270	225	3.324	1.2	2.780e^−11^
302	201	3.721	1.5	2.170e^−11^
349	174	4.299	2	1.514e^−11^

#### Constant acoustic energy density and wall displacement

**Figure 7:**
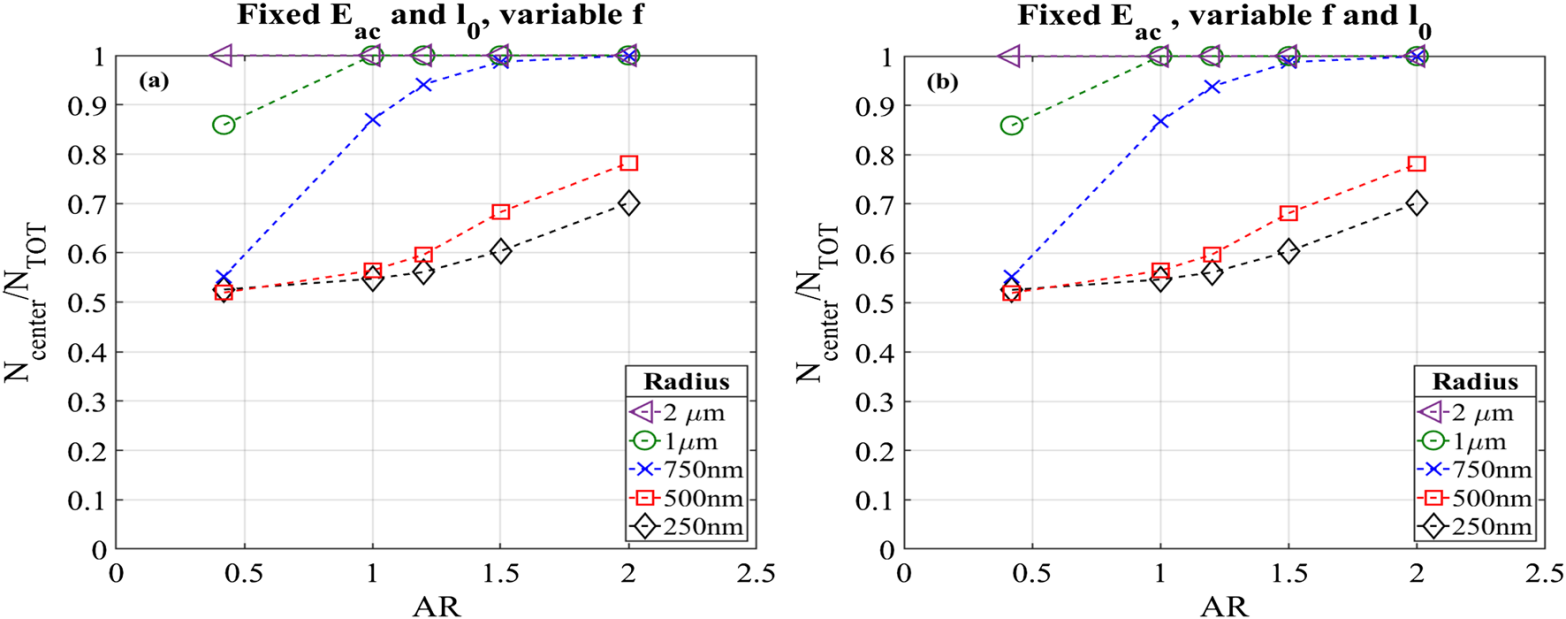
Fractions of particles in the central subdomain, compared to the total number released, as function of their radius. These results are recorded a t = 16 s after particles release. (**a**) The frequency was set to obtain an acoustic energy density of 106 Pa. (**b**) The frequency was changed for each AR to the resonance value and the displacement was varied obtaining an acoustic energy density constant.

Even in this case the excitation frequency was properly tuned to keep constant the acoustic energy density at around 106 Pa. Obviously, these values do not correspond to the resonance frequency, which leads to a tenfold increase in acoustic energy density. As in the previous section, we maintained 106 Pa to have a good comparison with the reference system and the frequencies used to remain at this value are reported in Table 4. The trends of the fraction of particles collected in the central domain are shown in Fig. 7a. For particles lower than 750 nm of radius, the situation does not change remarkably. On the other hand, for sizes equal and greater than this value, the percentages of particles collected in the central domain increase drastically, reaching 100% of focusing for the highest AR. This could be explained considering the mean path that a particle has to travel to achieve the central channel position. In fact, due to the smaller width of the channels, the focusing could be achieved in a shorter time. Particles with radius of 1 µm are collected in the center already for AR equal to 1. This is an interesting result with respect to the experiments described above, in which the same particles were collected only for 90% respect to the total number released.

#### Constant acoustic energy density at resonance frequency varying the wall displacement

As done in the case with a constant width, we also investigated the case in which the resonance frequency is applied, meanwhile the acoustic energy density is maintained at 106 Pa. The values of frequency and wall displacement used in this section are listed in Table 5. Similar data were obtained for these conditions compared to the one obtained with constant acoustic energy density and wall displacement. This could be considered as a validation to what was stated in the previous section. In fact, as reported in Fig. 7b, for smaller particles the focusing efficiency does not have a significant improvement, while for particles with a radius greater than 500 nm, the percentage in the center is increased from a 55% to 80%. The parameters which enhance the performance of the concentration efficiency are the cross section of the channel and the acoustic energy density. From these results, it is possible to underline the best conditions for achieving a good concentration of all particles in the center of the cross section. An increased aspect ratio, with a constant acoustic energy density and the cross-sectional area are kept constant, lead to a higher percentage of particle focused, also with particles of smaller radius that could be hard to concentrate when a common rectangular section is considered. In particular, comparing Figs. 6 and 7, this is highlighted for particles with radius greater than 500 nm, which are focused near at 100% with an AR of 1.2. Meanwhile, the 80% of particle with radius equal to or lower than 500 nm could be collected in the best case (Fig. 7a,b) of AR equal to 2. This improvement in the efficiency is reasonable if the balance of force acting on the particles is considered. From this point of view it is clear that in absence of acoustic streaming the only driving force is the acoustic radiation force that moves the particles (if they have a positive contrast factor) to the node of the standing wave. This means that the presence of a zone far from the boundaries, where the acoustic streaming does not affect the fluid motion, is useful to increase the percentage of focusing of particles.

## Conclusions

We numerically solved the isothermal acoustic problem in a microfluidic channel for the first mode of resonance. Stokes drag force and acoustic radiation force were calculated from the obtained fields and used to perform the particle tracing. To the best of our knowledge, we present the first systematic parametric study to analyze the effect of the aspect ratio on the ability to focus particles with diameter from 500 nm up to 4 µm due to acoustic streaming. Two different approaches were simulated and analyzed. The former was a study of different operating conditions in which the width of the channel was kept constant. The second was the same study but considering a constant S for all the aspect ratios. We show that in both the conditions with a constant width and cross-sectional area, a higher aspect ratio increased capability to efficiently focus particles, even with particles of smaller radius. This is due to the formation of a central “free zone” where the acoustic streaming does not influence the flow of the fluid and the acoustic radiation force is dominant. We observed also that working with a constant S is more efficient in terms of percentages of particles collected in the center of the channel. Any experimental studies were present in literature which could fit for our purpose. Unfortunately, the only reasonable data to consider are exposed in the already cited papers of Muller et al.^3,8^, but they are still referred a “common” rectangular section with aspect ratio lower than one. Instead of that our study aims to pave the way to the usage of different aspect ratios. We hope that this could trigger also other researcher to go further in this topic. Moreover, the experimental part of the work is certainly of interest to us, so it will be absolutely a topic for future works.

## Supplementary information

Supplementary Information.
