# Supplementary material for: Numerical study of the effect of channel aspect ratio on particle focusing in acoustophoretic devices
Source: Sci Rep. 2020 Nov 10;10:19447. doi: 10.1038/s41598-020-76367-w (PMC7655847; doi:10.1038/s41598-020-76367-w)
Supplement: Supplementary file 1 — Supplementary Information. [file 41598_2020_76367_MOESM1_ESM.docx]

**Numerical study of the effect of channel aspect ratio on particle focusing in acoustophoretic devices**

L. Spigarelli^1*^, N. S. Vasile^3^, C.F. Pirri ^1,2^, G. Canavese^1,2*^

^1^ Department of Applied Science and Technology, Politecnico di Torino, Corso Duca degli Abruzzi 24, 10129 Turin, Italy

^2^ Chilab - Materials and Microsystems Laboratory - DISAT Politecnico di Torino, Via Lungo Piazza d’Armi 6, 10034 Chivasso (Turin), Italy

^3^ SynBio lab, Italian Institute of Technology, Via Livorno 60, 10144 Torino.

corresponding author: [luca.spigarelli@polito.it](mailto:luca.spigarelli@polito.it), giancarlo.canavese@polito.it

**Supplementary Information**

**Number of particles affecting the results.**

**Figure S1.** Fraction of the particles collected in the central subdomain as function of time. The number of particles used goes from 270 to 6756. The aspect ratio used is 0.42 and the radius of the particles is 250 nm. These results were used for obtaining Fig. 3 in the paper.


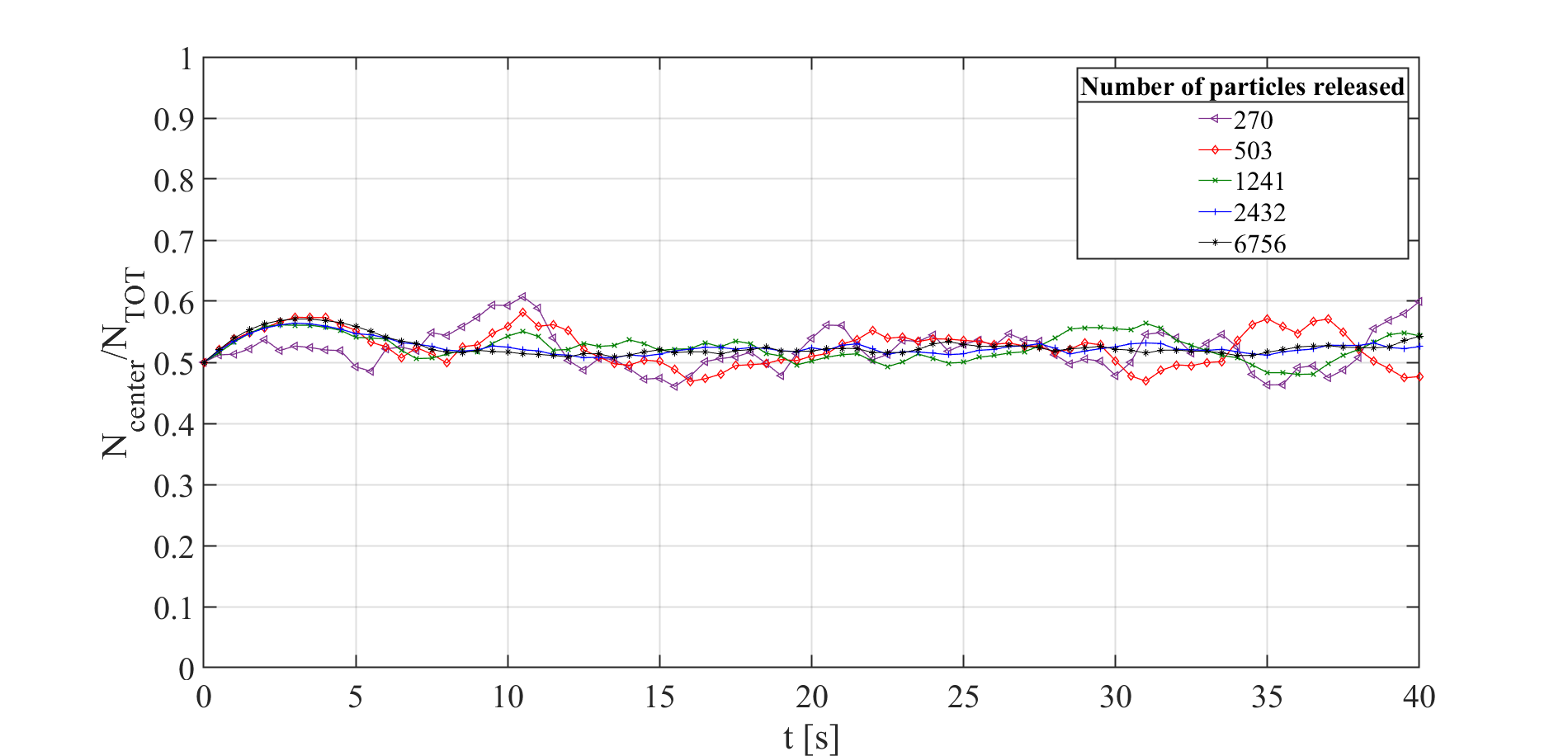


**Optimal focusing time**

**Figure S2.** Fraction of the particles collected in the central subdomain as function of time. The aspect ratio used varied from 0.42 to 2 and the radius of the particles is 250 nm. These results were used for finding an optimal time of focusing.


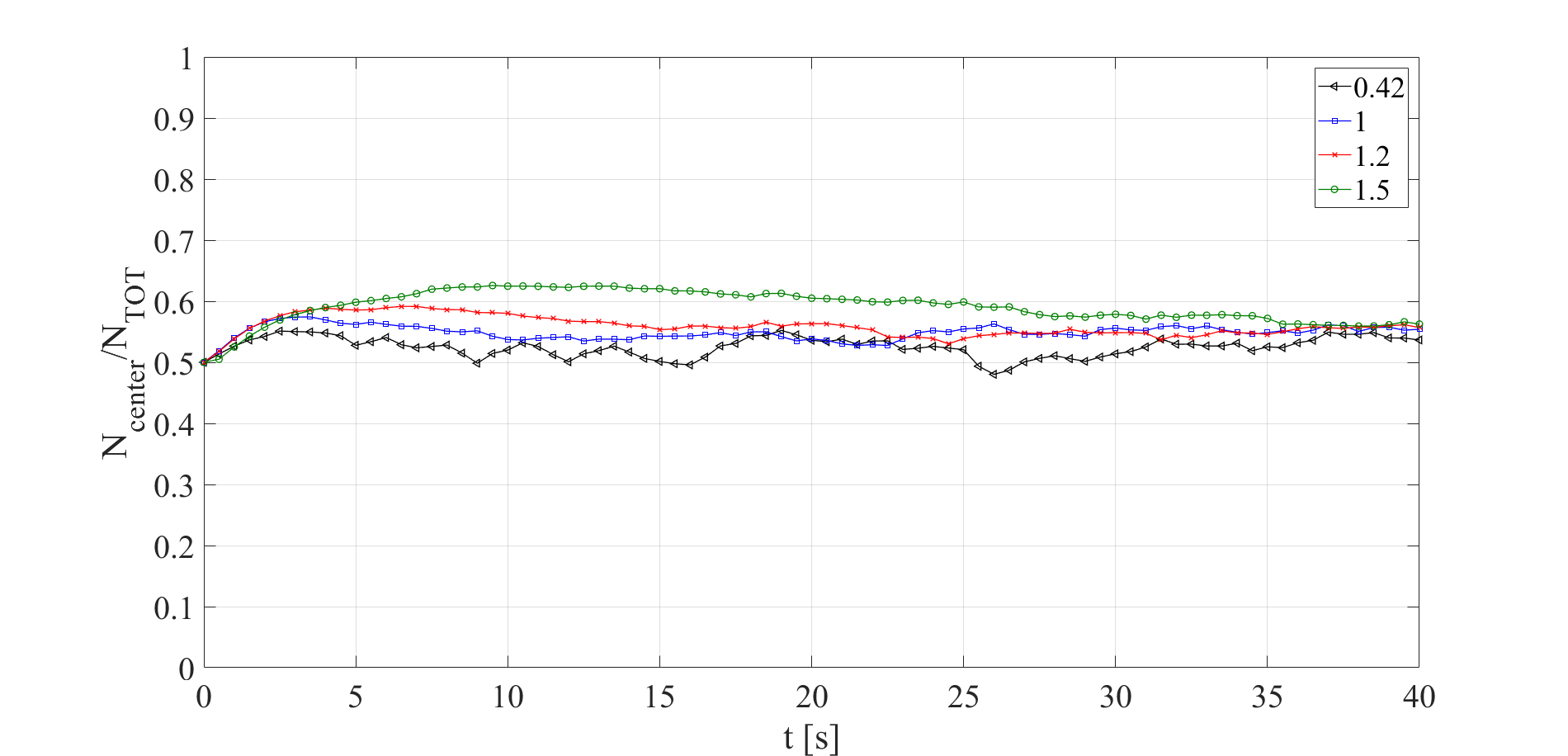


**Comparison 2D model with 3D model.**

A numerical 3D model applying the limiting velocity finite element method ^1,2^ ,was used to validate part of our results obtained with the 2D model proposed in the paper. Since many systems can be modelled appropriately using a 2D approximations, this comparison aims to understand the influence of the third dimension on the results. A full 3D numerical model which involves the resolution in the viscous boundary layer has heavy computational demand. Lei et al. developed a lighter 3D model ^2^ using the computationally efficient limiting velocity finite element method. This approach predicts the acoustic streaming only outside the viscous boundary layer, which means that the boundary-layer mesh is not needed reducing the computational effort. Thus, the limiting velocity theory was used to investigate the influence of the flowrate on the focusing results, in order to validate the proposed 2D model.

**Numerical method**

The first-order acoustic fields were simulated using the built-in COMSOL’s physics “Pressure Acoustics”. It solves the harmonic linearized equation which takes the form:

$\nabla^{2}p=-\frac{\omega^{2}}{c^{2}}p,$ (1)

where ω is the angular frequency, *p* is the harmonic pressure field and *c* is the speed of sound in water. We simulated the actuation of the device through displacement boundary conditions at the lateral walls.

Through the pressure field it is possible to solve the limiting velocity equations ^1^,

$u_{L}= - \frac{1}{4\omega}Re \left\{ q_{x}+ u_{1}^{*}\left[ (2+i)(\frac{du_{1}}{dx}+ \frac{dv_{1}}{dy}+\frac{dw_{1}}{dz})-(2+3i)\frac{dw_{1}}{dz} \right] \right\},$ (2)

$v_{L}= - \frac{1}{4\omega}Re \left\{ q_{y}+ u_{1}^{*}\left[ \left( 2+i \right)\left( \frac{du_{1}}{dx}+\frac{dv_{1}}{dy}+\frac{dw_{1}}{dz} \right)-\left( 2+3i \right)\frac{dw_{1}}{dz} \right] \right\},$ (3)

$q_{x}= u_{1}\frac{du_{1}^{*}}{dx} + v_{1}\frac{du_{1}^{*}}{dy},$ (4)

$q_{y}= u_{1}\frac{dv_{1}^{*}}{dx} + v_{1}\frac{dv_{1}^{*}}{dy},$ (5)

where $u_{1},v_{1}$ and $w_{1}$ are the x,y,z components of the first-order velocity field, $u_{L}$ and $v_{L}$ are the two components of the limiting velocities, and * represents the conjugate value.

For simulating the second-order fields, the predefined COMSOL’s “Creeping Flow” interface was used. A Stokes flow was considered; thus, the equations take the form,

$\nabla p_{2}=\mu\nabla^{2}\mathbf{v}_{\mathbf{2}},$ (6)

$\nabla\cdot\mathbf{v}_{\mathbf{2}}=0.$ (7)

The two limiting velocity equations were used as boundary conditions for the top and bottom walls, while a no-slip condition was set for the right and left walls. At the inlet (x=0) a low flow-rate condition is imposed, at (x=L) the pressure outlet condition was chosen.

The COMSOL ‘Particle Tracing for Fluid Flow’ interface was used to compute the particles trajectories. As written in the paper, the motion of the particles is computed taking in account of the balance between Stokes drag force and acoustic radiation force, we refer the reader to the main body of the paper.

**Geometry and Mesh**

The width and the height of the channel were chosen to be the same as in the paper, so 380 $\mu m$ and 160 $\mu m$ respectively. The length of the channel L was set at 2 cm. We chose to use a constant mesh element size of 50 $\mu m$, since 8-10 elements within the wavelength is enough for computing acoustic and streaming fields^1^. The 3D mesh obtained with COMSOL and used for the simulations is shown in Fig. S3.


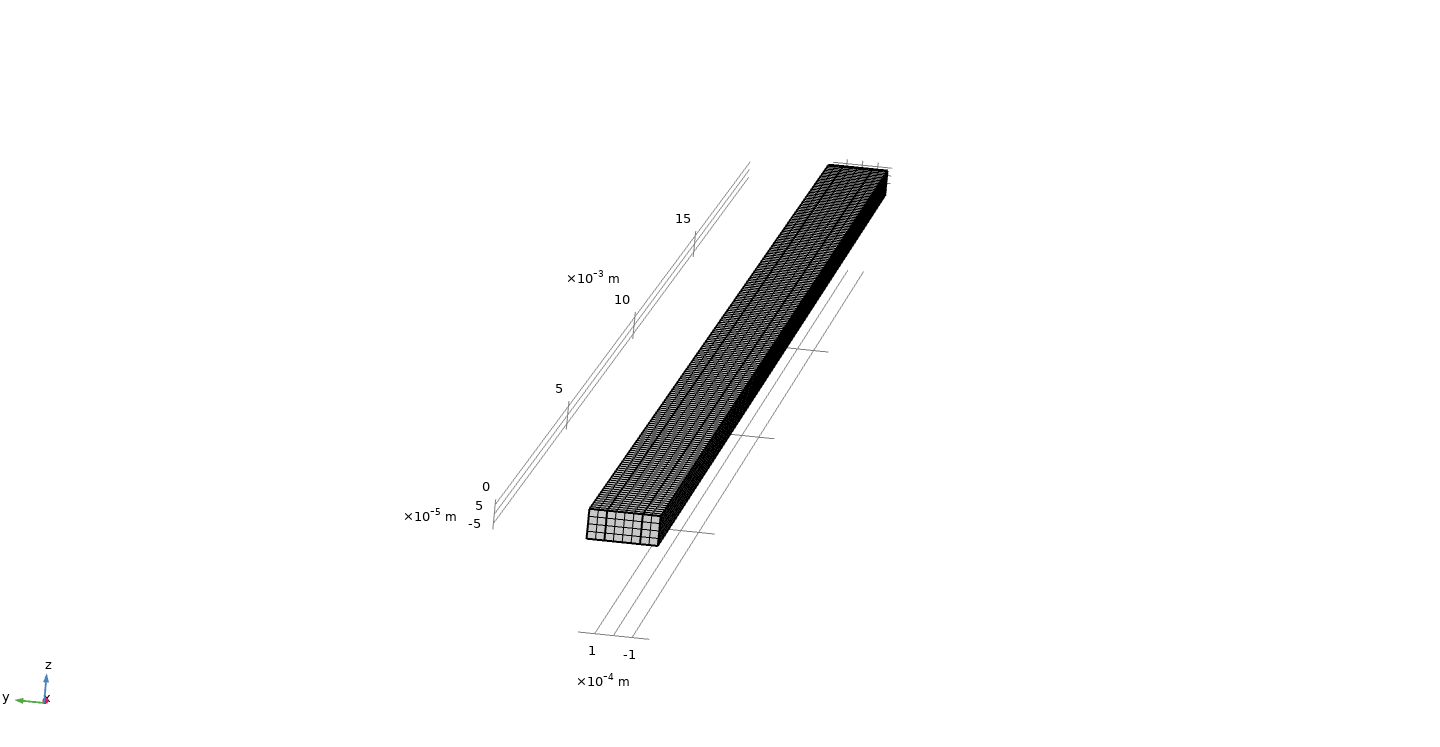

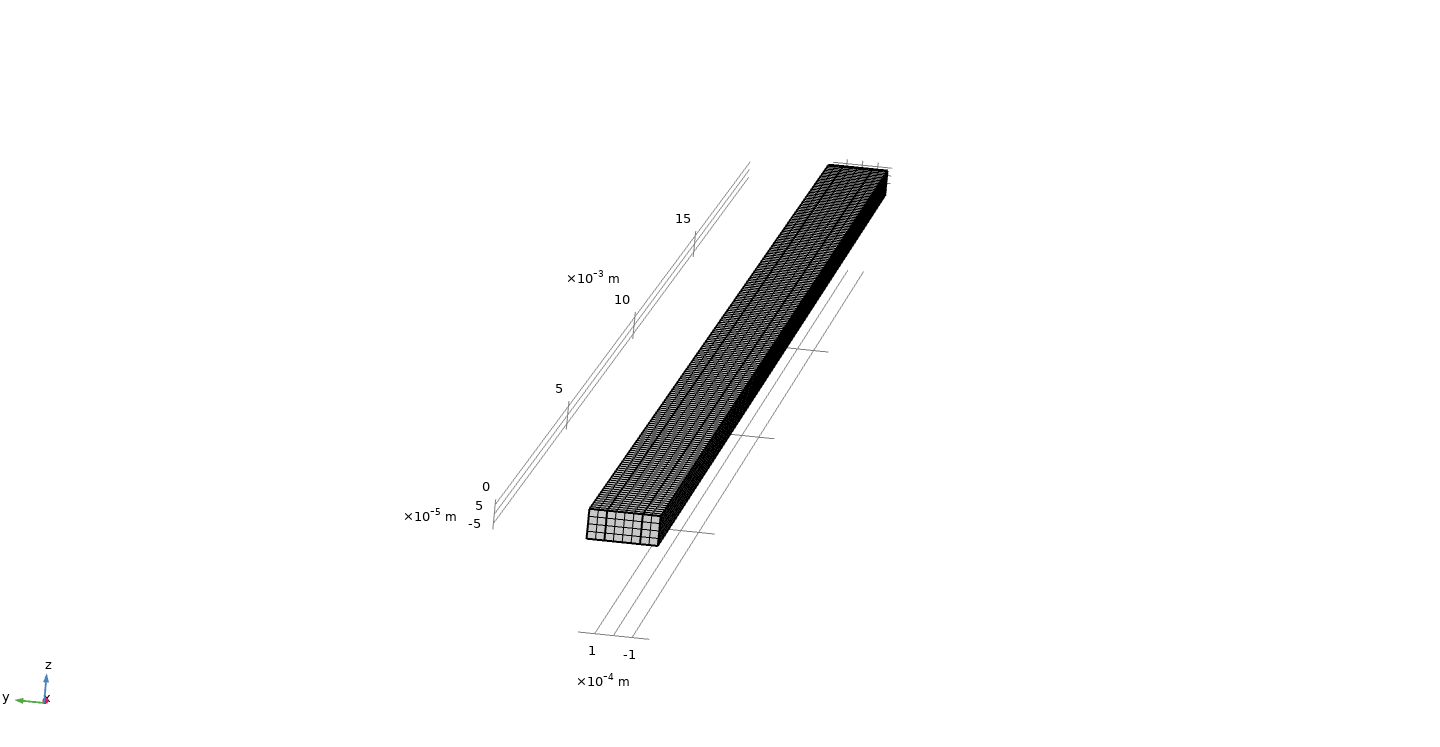


**Figure S3**. 3D channel with the mesh used in the simulations.

**Results**

We chose to simulate part of the conditions exposed in paper to have a comparison between the 2D proposed model and the 3D. First, we analysed the amplitude of acoustic streaming velocity and the first-order pressure field. As shown in Fig. S4, the magnitude are comparable. As it is possible to notice in Fig. S4, the difference between the fields is neglectable. Fig. S5 shows the first order pressure field in a 3D view. These results were obtained with an acoustic energy density of 106 Pa and with an actuation frequency of 1.9678 MHz. (in the 2D we used 1.9669 MHz, this little difference could be due by higher viscous damping in the 3D model that slightly shift the channel resonance). We also checked this by performing particle tracing analysis. As done in the paper, we computed the fraction of particles (with radius of 2 µm, 1 µm, 750 nm, 500 nm, and 250 nm) that can be collected in the central region of the cross-section. The results of the two models are reported in the table below. From the values we obtained, we can say that the general behaviour of the particles in not influenced by an imposed flow rate, such that the difference between the results from the 2D and 3D models is neglectable in this case.


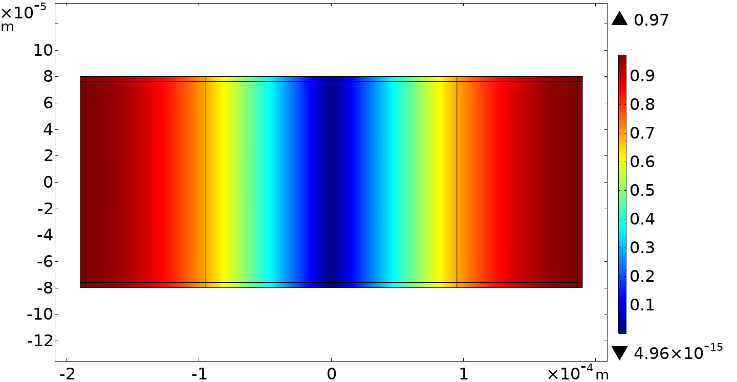

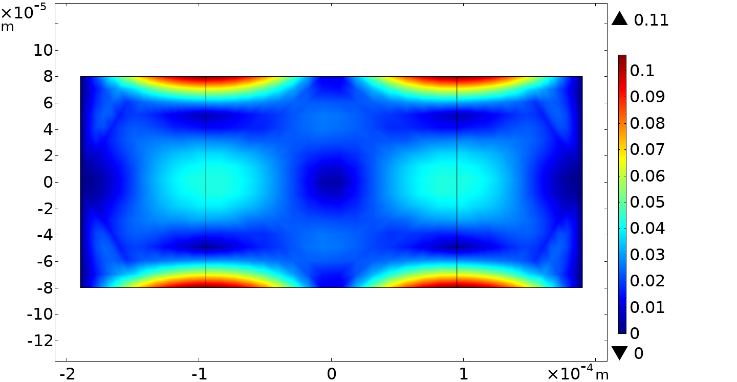

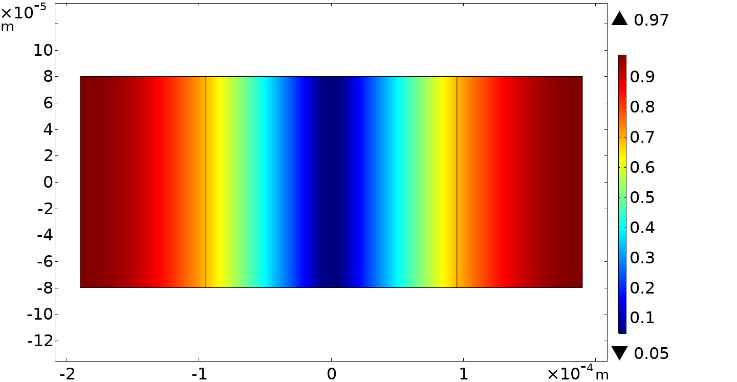


**Figure S4.** (a-b) Acoustic streaming velocity contour plot from 2D model and from 3D model, respectively. (c-d) First order pressure contour plot from 2D model and 3D model, respectively.


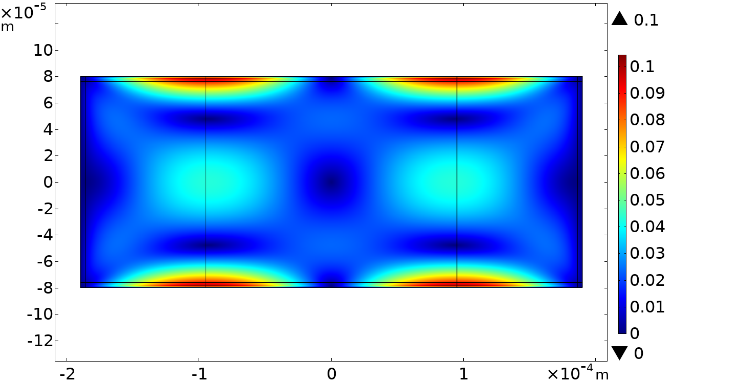


(a)

(b)

(c)

(d)

MPa

mm/s

mm/s

MPa


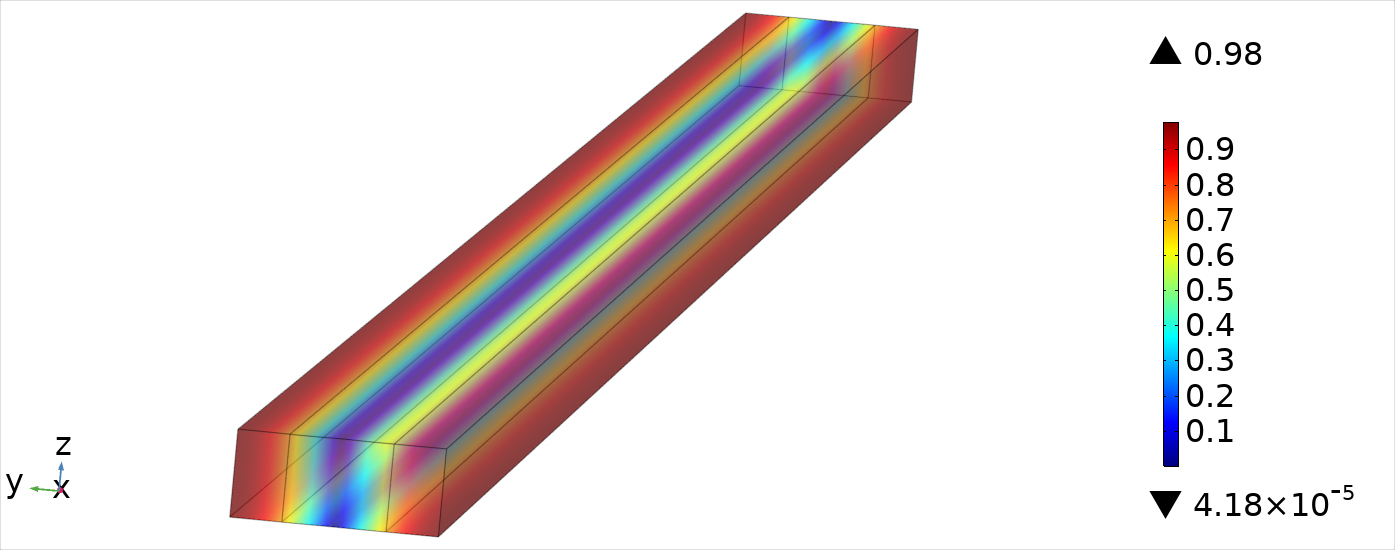


**Figure S5.** 3D plot of the first order acoustic field.

MPa

| **Table S1.** Results obtained from the particle tracing with the two models. | | | |
| --- | --- | --- | --- |
| **Radius** | **N_center_/N_sides_ from 2D model** | **N_center_/N_sides_ from 3D model** | **Deviation between the two models [%]** |
| 2 µm | 1 | 1 | 0 |
| 1 µm | 0.86 | 0.82 | 4.6 |
| 750 nm | 0.55 | 0.52 | 5.5 |
| 500 nm | 0.52 | 0.5 | 3.8 |
| 250 nm | 0.52 | 0.5 | 3.8 |
|  |  |  |  |

1. Lei, J., Hill, M. & Glynne-Jones, P. Numerical simulation of 3D boundary-driven acoustic streaming in microfluidic devices. *Lab Chip* **14**, 532–541 (2014).

2. Lei, J., Glynne-Jones, P. & Hill, M. Acoustic streaming in the transducer plane in ultrasonic particle manipulation devices. *Lab Chip* **13**, 2133–2143 (2013).
